# Monitoring immunomodulation strategies in type 1 diabetes

**DOI:** 10.3389/fimmu.2023.1206874

**Published:** 2023-06-06

**Authors:** Balasubramanian Krishnamurthy, Matthew Lacorcia, Thomas W. H. Kay, Helen E. Thomas, Stuart I. Mannering

**Affiliations:** ^1^ Immunology and Diabetes Unit, St Vincent’s Institute, Fitzroy, VIC, Australia; ^2^ Department of Medicine, St Vincent’s Hospital, University of Melbourne, Fitzroy, VIC, Australia

**Keywords:** type 1 diabetes, biomarkers, T cell exhaustion, antigen specific therapy, disease modifying treatment

## Abstract

Type 1 diabetes (T1D) is a T-cell mediated autoimmune disease. Short-term treatment with agents targeting T cells, B cells and inflammatory cytokines to modify the disease course resulted in a short-term pause in disease activity. Lessons learnt from these trials will be discussed in this review. It is expected that effective disease-modifying agents will become available for use in earlier stages of T1D. Progress has been made to analyze antigen-specific T cells with standardization of T cell assay and discovery of antigen epitopes but there are many challenges. High-dimensional profiling of gene, protein and TCR expression at single cell level with innovative computational tools should lead to novel biomarker discovery. With this, assays to detect, quantify and characterize the phenotype and function of antigen-specific T cells will continuously evolve. An improved understanding of T cell responses will help researchers and clinicians to better predict disease onset, and progression, and the therapeutic efficacy of interventions to prevent or arrest T1D.

## 1 Introduction

In Type 1 Diabetes (T1D), loss of immune tolerance allows autoreactive T cells to destroy the insulin-producing beta cells in the pancreatic islets (1). Successive failures in self-regulatory checkpoints are required to both achieve and perpetuate this end-stage of immunopathology.

For over 100 years, insulin has been the sole effective treatment for T1D. Insulin-based treatments are complex, costly, and limited by the risk of hypoglycaemia (2). Knowledge gained from cumulative work over many years has helped us to understand the process leading to the beta-cell’s demise. Recognizing the contribution of human leukocyte antigen (HLA) genes (and other genes) to the risk of developing T1D (3, 4), as well as defining the natural history of islet autoantibodies in subjects at risk for T1D (5), has enabled the staging of pre-symptomatic T1D into four stages Stage 0 (or Pre-Stage 1), genetic risk, Stage 1, development of islet autoantibodies, Stage 2, development of prodromal metabolic abnormalities and finally, Stage 3, onset of clinical symptoms (6). The classification of T1D stages highlight the development of beta cell autoimmunity and beta cell loss that occurs prior to the onset of clinical disease. This framework now allows a more targeted disease-modifying therapy based on the patient’s disease stage.

Both CD4+ and CD8+ T cells participate in the pathogenesis of T1D (7, 8). CD4+ T cells are implicated because of the strong association of T1D with HLA class II, specifically the haplotypes HLA-DR4-DQ8 and HLA-DR3-DQ2. Islet antigen specific CD4+ T cells, restricted by HLA-DR4, DR3, DQ2 and DQ8, have been detected in the blood and pancreatic islets of organ donors who had T1D patients (8–13). CD4+ T cells provide ‘help’ to B and CD8+ T cells. CD8+ T cells reactive to islet antigens are the predominant T cells in insulitis and they mediate beta cell killing in T1D (14–17). As the diagnosis of T1D approaches, T cells reactive to numerous islet autoantigens undergo clonal expansion (11, 18–23). These are long-lived, self-renewing memory CD8+ T cells and have a capacity to differentiate into effector T cells (15, 24, 25). The challenge of reversing pathogenic immune responses in T1D requires not only directed therapy directed against effector T cells, but also prevention of reactivation of memory responses when therapy is discontinued.

Over the last three decades, an array of immune therapeutics targeting T cells (anti-thymocyte globulin and antibodies targeting CD2, CD3 and CD80/CD86), B cells (antibody targeting CD20), and cytokines (IL-1, IL-2, IL-6, IL-12/IL-23, IL-21, TNF-a) have been evaluated in clinical trials for efficacy in altering the course of T1D (26, 27). The results of many such trials indicate that the immune therapies can preserve or slow the loss of beta cell function for a short period of time [reviewed in (27)]. But no long-lasting maintenance of C-peptide was achieved. However, despite not achieving their ultimate goal of long-term retention of C-peptide, many important lessons have been learnt from the mechanistic findings of these trials. In this review, we aim to address how we could apply the knowledge gained by the mechanistic studies of the clinical trials in monitoring immunotherapy for T1D.

## 2 Antigen-specific therapy

It has long been thought that the limitations of current therapies could potentially be addressed by the introduction of antigen-specific approaches. These strategies typically require administration of autoantigenic proteins, or peptides, or autoantigen-encoding nucleic acids. Although the mechanisms have not been fully elucidated, these approaches generally operate by functionally inactivating and/or deleting cognate autoreactive T cells (28–31). Various forms of (pro)insulin (32–38) and GAD65 (39–43) have been administered in clinical trials, with the goal of maintaining residual endogenous beta cell function in stage 3, or with the goal of preventing the progression to stage 3 of disease in individuals with stage 1 and stage 2 disease. None of the antigen-specific therapy clinical trials delayed or stopped the autoimmune process preceding onset of T1D. Proinsulin and proinsulin DNA preserved C-peptide transiently. Treatment with GAD did not consistently meet primary efficacy endpoints in recent onset T1D subjects (39, 44). Antigen-specific therapies induced a myriad of different responses including increased (45, 46) or blunted (38) antigen specific antibody response, increased (46, 47) or decreased (45, 48) T cell proliferative response, decrease in antigen induced IFNγ (38), increase in TGFβ (47), IL-10 (49) or Th2 cytokine response (50), increased regulatory T cells (51) and decreased antigen specific CD8+ T cells (32). Thus, from trials that characterized the T-cell response, it is difficult to understand the reason for failure to achieve therapeutic benefit. A set of validated biomarker assays focused on the quantity and quality of islet antigen-specific T cells is needed to assess directly whether candidate therapies are achieving their mechanistic goals (52).

## 3 Preclinical antigen-specific therapies

Mechanistic studies in NOD mice, a spontaneous model for T1D, have helped us to understand aspects of antigen therapy in the context of autoimmune diabetes. In NOD mice proinsulin is the driver antigen for diabetes. Disabling proinsulin specific immune response by either deletion of proinsulin antigen (53) or deletion of proinsulin specific T cells prevents diabetes (29, 54). In contrast, a similar approach with GAD-65 (28, 55), IA-2 (56) or IGRP (30, 57, 58) did not prevent diabetes in NOD mice (28–30, 53–58). This indicates that proinsulin occupies a key position in the autoimmune responses against beta cells. As the disease progresses ‘epitope spreading’ occurs, when the number of antigens targeted by T cells increases (30). It is therefore not surprising that functional inactivation, or deletion, of T cells targeting disease-initiating autoantigens can blunt disease progression following early intervention, yet fail to have an impact at more advanced stages of disease (59), as is usually the case in human clinical trials. In order for antigen-specific therapies against T1D to be successful, treatment should induce T cells that can suppress immune responses to as wide an array of islet antigens as possible (60). Importantly, any antigens specific suppression should be measurable to determine if ongoing treatment is required to maintain immune tolerance status. We previously showed that deletion of IGRP specific T cells by expressing IGRP in the antigen presenting cells from birth did not protect NOD mice from diabetes (30). Expression of IGRP in the antigen presenting cells after 10 weeks of age when the IGRP-specific T cells have increased in number and developed into memory T cells after encountering antigen in the islet induced exhaustion of IGRP- specific T cells instead of deleting them (31). NOD mice can be protected from diabetes at a time when an immune response is established against multiple antigens by transgenic IGRP expression via inducing exhaustion in T cells specific for IGRP (31). This suggests a dominant tolerance mechanism i.e. that dominant tolerance to IGRP-inhibits T cells specific for other islet antigens. Dominant tolerance with protection from diabetes in NOD mice have also been noted with subcutaneous liposomal co-delivery of IGRP peptide with a Vitamin D3 analogue and nanoparticle coated with IGRP peptide-MHC class I complexes (61). These results show that inducing T-cell exhaustion rather than deletion of antigen-specific T cells is desirable.

## 4 T-cell exhaustion and T-cell regulation

T-cell exhaustion is a state of dysfunction in CD8+ T cells that develops during chronic antigen exposure. It is characterized by loss of functional capabilities such as cytokine production, cytotoxicity and proliferative capacity. Following stimulation, exhausted T cells have reduced capacity to generate effector CD8+ T cells and these T cells have decreased effector function. Exhausted T cells express multiple co-inhibitory receptors (e.g. CTLA-4, PD-1, LAG-3, TIM3, and TIGIT) and respond poorly to cytokines resulting in less long-term survival (62, 63). While T cell exhaustion has been described in CD4+ T cells, the field is still evolving. T cell exhaustion in CD8+ T cells is well established and hence we will focus the review on CD8+ T cell exhaustion.

Tumors and chronic viral infections exploit T cell exhaustion to avoid clearance by effector mechanisms. The exhausted phenotype results from a differentiation process in which T cells stably adjust their proliferative and effector capacity to a lower level and this phenotype is optimized to cause minimal tissue damage while still mediating a critical level of pathogen or tumor control. Exhausted T cells are not only hypofunctional but also upregulate molecules that suppress local T cell immunity such as CD39 and IL-10. A recent study has shown that CD39 expressing exhausted cells have suppressive capacity similar to conventional FoxP3 expressing CD4+ regulatory T cells (64). Several reports have identified that IL-10 secreting CD8+ T cells play a regulatory role in protecting against autoimmune disease (65). Conversely, blocking of IL-10 signaling improved the function of exhausted T cells in chronic viral infections (66) and simultaneous blockade of IL-10 and PD-1 pathways resulted in elimination of persistent viral infection (67).

Pathways related to T-cell exhaustion play an important role in restraining T cells in autoimmunity. It takes months (in mice) to years (in humans) after onset of autoimmunity to develop diabetes. In contrast, autoimmune diabetes is rapidly induced by blocking the PD-1 pathway in NOD mice (68). We and others have shown that rapid onset of T1D follows checkpoint inhibition in humans with pre-existing islet autoimmunity (69, 70). Recent studies have identified exhausted T cells in islets of NOD mice (71–73). Transcriptomic profiling of T cells from patients with autoimmunity showed that a T cell exhaustion signature correlated with a more benign form of autoimmune disease, indicating that mechanisms associated with T cell exhaustion may be important in controlling autoimmunity (74). Following teplizumab (anti-CD3) and alefacept (LFA-3_Ig fusion protein) treatment, subjects with a greater proportion of exhausted islet specific CD8+ T cells demonstrated slower progression of T1D (75, 76).

## 5 Lessons from clinical trials

Clinical trials using a broad range of immunotherapy strategies in new-onset T1D resulted in preservation of insulin secretory capacity. This pattern has been true for therapies that target different components of the immune response, including B-cell depletion (rituximab, anti-CD20) (77, 78), co-stimulation blockade (abatacept, CTLA4-Ig fusion protein) (79, 80), T cell depletion with low-dose anti-thymocyte globulin (2.5 mg/kg) (81) or suppressing T cell function with anti-CD3 (using teplizumab or otelixizumab) (82–85) and anti-CD2 (alefacept) (86, 87). Similar effects were also achieved using therapies directed against cytokines that contribute to the inflammatory islet environment, to inhibit the processes that support effector or memory T cells and suppress regulatory T cells. Inhibition of TNF-α (using the antibodies etanercept or golimumab) (88, 89) and anti-IL-21 (90), for example, resulted in preservation of C-peptide, although no benefit was seen with IL-6 receptor blockade (tocilizumab) (91) or IL-1β blockade (canakinumab) (92). An important question regarding approaches that target cytokines is whether blocking individual cytokines is sufficient, given the redundancy in cytokine pathways. The aim of treatments that target cytokines is to push the antigen experienced T cells towards a regulatory and anti-inflammatory pathway. However, it is not known whether the autoantigen exposure that naturally occurs in islets in T1D is sufficient to push towards regulatory/anti-inflammatory pathways given the preclinical data suggesting most of the islet reactive T cells reside in peripheral lymphoid organs (31) and minimal antigen exposure in the islets by the time of diagnosis of T1D. It is quite possible that it will be necessary to provide additional antigen, or TCR stimulation with anti CD3 antibody, simultaneously with cytokine blockade for the desired long-term response.

Until recently, the lack of sophisticated technologies had precluded deep analyses of T-cell subsets to represent meaningful immune alterations for clinical contexts. The use of MHC multimers and mass cytometry to phenotype T cells in recent trials has helped us better understand phenotypic and functional changes following treatment. T lymphocytes drive many arms of the immune response. T1D evolves over many years and this chronicity is likely to be due to a balance between the autoimmune attack and processes such as T cell regulation via Treg and T cell exhaustion, that reduce its effectiveness. Immunotherapy can alter this balance as has been shown in some of the recent clinical trials. Anti-thymocyte globulin in higher dose (6.5 mg/kg) was used to deplete cellular immune effector compartments as an attempt to arrest immune-mediated damage to beta cells (93). T-cell depletion was highly effective, however, there was also loss of regulatory T cells resulting in no alteration of the balance of effector and regulatory arms. There was no metabolic benefit from drug administration. With anti-CD3 antibody there was metabolic benefit (94, 95) and deep analysis of immune biomarkers revealed upregulation of markers of T cell exhaustion in CD8 T cells (76, 96). Instead of T cell depletion, anti CD3 treatment induced a developmental program towards a regulatory exhaustion phenotype. This pathway was also noted with anti-CD2 treatment and was associated with beneficial clinical outcome (75). While anti-CD3 and anti-CD2 were developed for inducing T cell deletion, clinical response was correlated with T cell exhaustion. This suggests that induction of an exhaustion phenotype rather than depletion of T cells is desirable for the best outcome. This is consistent with our data in NOD mice (31). Treatment with low dose IL-2 to boost regulatory T cells in recent onset T1D resulted in expected expansion of regulatory T cells in treated subjects but the trial was terminated early due to unexpected rapid loss of C-peptide that implied potential acceleration of disease (97). Biomarker studies of these subjects documented no changes in T effector or memory cells but an increase in CD56hi NK effector cells due to IL2 stimulatory effect, counterbalancing the increased regulatory T cells.

## 6 Biomarkers

Biomarkers have potential applicability in multiple disease phases of T1D. Biomarkers could be used to predict disease onset, disease progression, stratify patients for appropriate treatment, and to monitor effect of treatment. Biomarkers could be particularly useful in determining the response to pharmacological treatment. Clinical trials in T1D measure C-peptide and various glycaemic parameters (HbA1c, glucose time in range, glycemic variability on continuous glucose monitoring) to provide an indirect assessment of beta-cell function. Measuring changes in the number and phenotype of immune cell subsets, as well as more specifically monitoring changes in (a) the immune populations that are directly related to the drug mechanism of action (eg, depletion of T cells with anti-thymocyte globulin or increasing in regulatory T cells with IL-2 treatment) and (b) the phenotype and function of islet antigen-specific T cells in response to treatment will be important for improving clinical trials. This information will also guide subsequent approaches to therapeutic interventions. There are excellent recent reviews on factors impeding progress toward the development of effective T-cell biomarkers in T1D (52, 98, 99).

Major assays in current use to identify and quantify islet-reactive T cells include measuring proliferation via or dye-dilution such as the CFSE-based proliferation assay (100), upregulation of activation markers upon stimulation (101), or measuring cytokine secretion (19) (eg ELISpot) following stimulation with islet associated protein or peptide. The underlying challenge of faced by all assays is the very low frequency of antigen specific T cells in the peripheral blood and small quantity of blood available for analysis particularly from children. The benefit of these assays is that reactivity to multiple different antigens, or epitopes, may be tested simultaneously, and the assay is not restricted to individuals with a particular HLA. However, detection depends on the ability of the T cells to respond to the stimuli by proliferation or cytokine production. With ELISPOT assay only a limited number of cytokines is measurable in a single assay. Quantifying multiple cytokines in stimulated cell assay supernatants using multiplex immunoassays (such as Luminex) has been used, but such approaches do not quantify the proportion of responding cells.

A more direct way to analyze T cells is by assessing the binding to labelled major histocompatibility complex (MHC)–peptide multimers. Using peptide–MHC multimers, the frequency of antigen specific T cells along with their phenotype and function can be analyzed, but the scope is reduced by technical limits to relatively small numbers of target peptides and HLA types. A limited number of parameters can be analyzed using standard flow cytometry and the number of parameters (up to 40) can be increased using spectral flow cytometry or mass cytometry. Using single-cell mass cytometry along with a combinatorial pooled peptide–loaded MHC tetramer staining approach, CD8+ T cell number, function and phenotype was assessed in the peripheral blood T1D subjects (76). Islet antigen specific T cells expressed CXCR3 indicating that these cells were activated and enroute to islets as inflamed islets express its ligand CXCL10 (102–104). Islet antigen specific T cells were phenotypically heterogenous but enriched either in memory or exhausted phenotypes. Memory phenotype of islet-specific T cells were more frequent in in subjects with rapid decline in C-peptide, whereas an exhausted phenotype was more prevalent in subjects whose C-peptide level was preserved or declined slowly. The exhaustion phenotype was confirmed functionally in that they proliferated less to stimulation.

The use of combinatorial multimers and mass cytometry has enabled deeper understanding into cell phenotype and antigen specificity in T1D (75, 76, 105, 106). They allow detection of up to 40-50 of predetermined markers. While this gives a thorough phenotypic picture of the cells it may limit the potential to discover novel markers that change with the treatment and determine the outcome of the treatment. There are challenges in ex vivo assays on peripheral blood mononuclear cells (52) such as low frequency of autoreactive T cells, high receptor diversity of autoreactive T cells, limited known T cell epitopes, low volume of blood for analysis and analysis of T cells usually limited to peripheral blood but islet antigen-specific T cells reside predominantly in the lymph nodes (and islets). Analysis of T cells from the islets and novel approach to identify neo-epitopes targeted by T cells (11, 13, 23) will make it possible to capture antigen-specific T cells more widely. These assays can be refined by information from comprehensive and in-depth analysis and with unbiased approach such as scRNA-seq and single-cell TCR-seq to assess the clinical significance of T cells. For the immunomodulatory agents used in clinical trials for T1D, it is important to discover the pathways that change specifically in the islet antigen specific T cells following treatment. Single cell RNA sequencing (scRNA-seq) through the identification of genes that are differentially expressed in T cells and other immune cells following treatment has potential to discover novel outcome determining markers after immunotherapy. Combining the use of combinatorial peptide MHC multimers and surface antibodies with scRNA-seq (cellular indexing and Transcriptomes and Epitopes by sequencing, Cite-seq) allows simultaneous capture of cell surface protein and mRNA expression of single cells allowing optimal annotation of cell populations and identification of rare islet antigen specific T cells (107, 108). Cite-seq allows multiplexing the pre, during and after treatment samples using cell hash-tagging to reduce the batch effects and costs. The recovered TCR sequences can be used to determine the T-cell clonality of multimer positive cells (107). This approach can be used to define distinct cell states and their molecular circuitry of rare antigen specific T cells and then link these features with distinct disease outcomes and has potential to help us understand the mechanisms that drive the disease pathology.

## 7 Future directions

**Figure 1:**
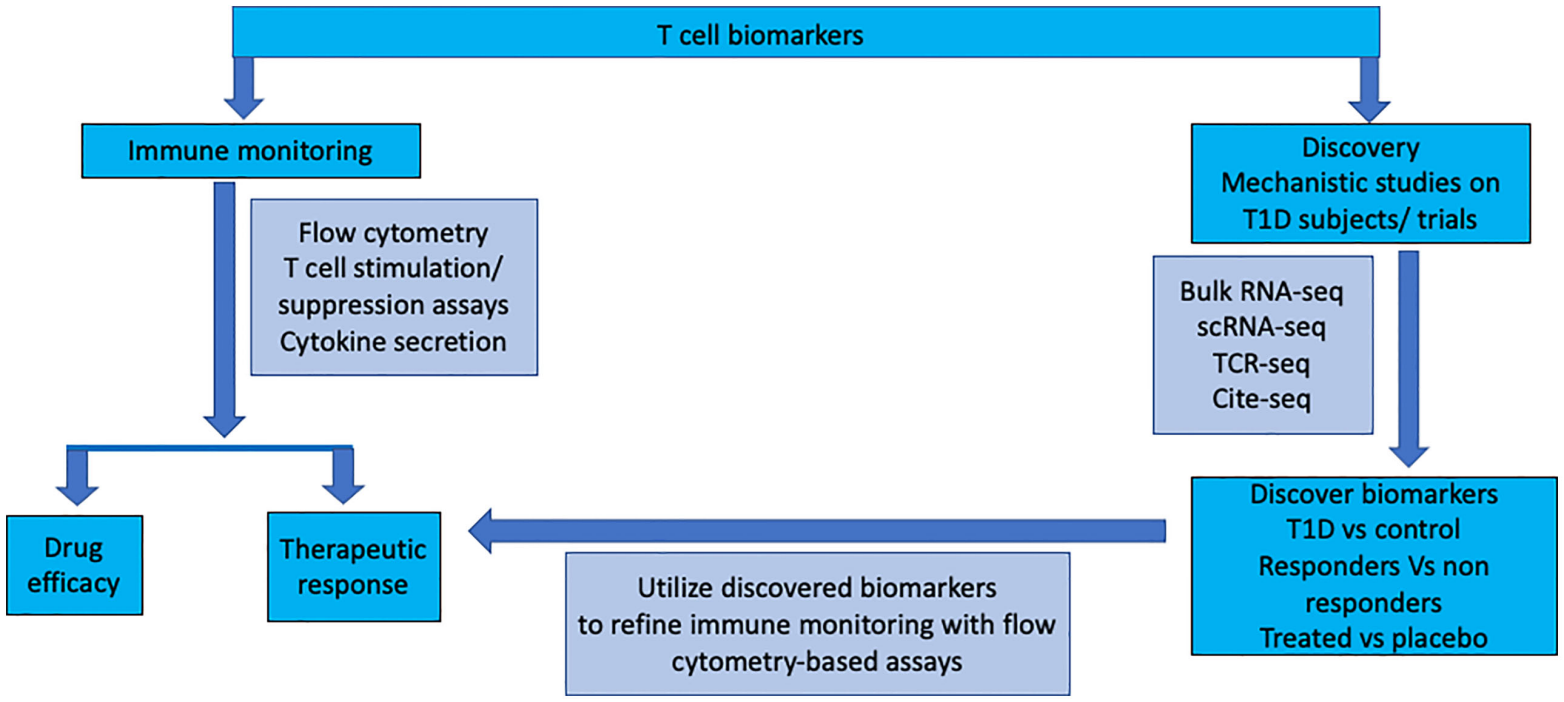
T cell monitoring during immune intervention in type 1 diabetes: *Ex vivo* assays on peripheral blood mononuclear cells are utilised for assessing drug efficacy and monitoring therapeutic response in clinical intervention trials. Basic research to identify neo-epitopes targeted by T cells will make it possible to capture antigen-specific T cells more widely. High-dimensional analysis of gene, protein and TCR expression will identify novel markers expressed on T cells during different stages of the disease and in response to treatment. This information will be utilised to refine ex vivo peripheral blood analysis to detect, quantify, and characterise T cell populations.

Clinical trials in T1D give us the opportunity to perform in-depth mechanistic studies to understand the disease process. The aim of the mechanistic study is to discover biomarkers and pathways in disease pathogenesis by unbiased analysis of proteins and genes in the relevant cells using modern technology. The discovered biomarkers can be further validated and applied to immune monitoring in further studies and clinical follow up using standard flow cytometry (**Figure 1**). The modern technology has just begun to be utilized in clinical trials of T1D. Further understanding the requirement of samples (processing time, transportation temperature of blood samples and effect of cryopreservation) for the modern assays is required. Also, the effect of age, pubertal status and different phases of disease on the expression of proteins and genes in the cells need to be understood for proper analysis and interpretation of the data. The cells are analyzed from peripheral blood, but cells from lymph nodes, spleen and islets should also be analyzed from T1D organ donors and compared to cells from peripheral blood. In T1D frequency of antigen specific T cells is low, the T cell-antigen interaction is low affinity, only a limited number of T-cell epitopes have been discovered and there is vast TCR diversity for a given epitope. All these factors make detection of antigen specific T cells difficult. Technological innovations in epitope discovery will allow multimer generation [eg., spheromers (109) to improve T cell- antigen interaction particular incorporating hybrid peptides (13)] for the identification of antigen-specific T cells and increase the understanding of T-cell correlates of disease protection.

## Author contributions

BK, ML, TK, HT and SM wrote the manuscript. BK produced the figure. All authors contributed to the article and approved the submitted version.

## References

[1] Mannering SI Pathiraja V Kay TW. The case for an autoimmune aetiology of type 1 diabetes. Clin Exp Immunol (2016) 183(1):8–15. doi: 10.1111/cei.12699 26313217 PMC4687512

[2] Foster NC Beck RW Miller KM Clements MA Rickels MR DiMeglio LA. State of type 1 diabetes management and outcomes from the T1D exchange in 2016-2018. Diabetes Technol Ther (2019) 21(2):66–72. doi: 10.1089/dia.2018.0384 30657336 PMC7061293

[3] Barbosa J Chern MM Anderson VE Noreen H Johnson S Reinsmoen N. Linkage analysis between the major histocompatibility system and insulin-dependent diabetes in families with patients in two consecutive generations. J Clin Invest (1980) 65(3):592–601. doi: 10.1172/JCI109704 6766467 PMC371400

[4] Erlich H Valdes AM Noble J Carlson JA Varney M Concannon P. HLA DR-DQ haplotypes and genotypes and type 1 diabetes risk: analysis of the type 1 diabetes genetics consortium families. Diabetes (2008) 57(4):1084–92. doi: 10.2337/db07-1331 PMC4103420 18252895

[5] Ziegler AG Rewers M Simell O Simell T Lempainen J Steck A. Seroconversion to multiple islet autoantibodies and risk of progression to diabetes in children. Jama (2013) 309(23):2473–9. doi: 10.1001/jama.2013.6285 PMC4878912 23780460

[6] Insel RA Dunne JL Atkinson MA Chiang JL Dabelea D Gottlieb PA. Staging presymptomatic type 1 diabetes: a scientific statement of JDRF, the endocrine society, and the American diabetes association. Diabetes Care (2015) 38(10):1964–74. doi: 10.2337/dc15-1419 PMC5321245 26404926

[7] Pugliese A. Autoreactive T cells in type 1 diabetes. J Clin Invest (2017) 127(8):2881–91. doi: 10.1172/JCI94549 PMC5531393 28762987

[8] Babon JA DeNicola ME Blodgett DM Crevecoeur I Buttrick TS Maehr R. Analysis of self-antigen specificity of islet-infiltrating T cells from human donors with type 1 diabetes. Nat Med (2016) 22(12):1482–7. doi: 10.1038/nm.4203 PMC5140746 27798614

[9] Mannering SI Dromey JA Morris JS Thearle DJ Jensen KP Harrison LC. An efficient method for cloning human autoantigen-specific T cells. J Immunol Methods (2005) 298(1-2):83–92. doi: 10.1016/j.jim.2005.01.001 15847799

[10] Pathiraja V Kuehlich JP Campbell PD Krishnamurthy B Loudovaris T Coates PT. Proinsulin-specific, HLA-DQ8, and HLA-DQ8-transdimer-restricted CD4+ T cells infiltrate islets in type 1 diabetes. Diabetes (2015) 64(1):172–82. doi: 10.2337/db14-0858 25157096

[11] So M Elso CM Tresoldi E Pakusch M Pathiraja V Wentworth JM. Proinsulin c-peptide is an autoantigen in people with type 1 diabetes. Proc Natl Acad Sci U States America (2018) 115(42):10732–7. doi: 10.1073/pnas.1809208115 PMC6196477 30275329

[12] Kent SC Chen Y Bregoli L Clemmings SM Kenyon NS Ricordi C. Expanded T cells from pancreatic lymph nodes of type 1 diabetic subjects recognize an insulin epitope. Nature (2005) 435(7039):224–8. doi: 10.1038/nature03625 15889096

[13] Delong T Wiles TA Baker RL Bradley B Barbour G Reisdorph R. Pathogenic CD4 T cells in type 1 diabetes recognize epitopes formed by peptide fusion. Science (2016) 351(6274):711–4. doi: 10.1126/science.aad2791 PMC4884646 26912858

[14] Coppieters KT Dotta F Amirian N Campbell PD Kay TW Atkinson MA. Demonstration of islet-autoreactive CD8 T cells in insulitic lesions from recent onset and long-term type 1 diabetes patients. J Exp Med (2012) 209(1):51–60. doi: 10.1084/jem.20111187 22213807 PMC3260877

[15] Skowera A Ellis RJ Varela-Calvino R Arif S Huang GC Van-Krinks C. CTLs are targeted to kill beta cells in patients with type 1 diabetes through recognition of a glucose-regulated preproinsulin epitope. J Clin Invest (2008) 118(10):3390–402. doi: 10.1172/JCI35449 PMC2542849 18802479

[16] Bulek AM Cole DK Skowera A Dolton G Gras S Madura F. Structural basis for the killing of human beta cells by CD8(+) T cells in type 1 diabetes. Nat Immunol (2012) 13(3):283–9. doi: 10.1038/ni.2206 PMC3378510 22245737

[17] Rodriguez-Calvo T Krogvold L Amirian N Dahl-Jorgensen K von Herrath M. One in ten CD8(+) cells in the pancreas of living individuals with recent-onset type 1 diabetes recognizes the preproinsulin epitope PPI(15-24). Diabetes (2021) 70(3):752–8. doi: 10.2337/db20-0908 PMC7897350 33414250

[18] Martinuzzi E Novelli G Scotto M Blancou P Bach JM Chaillous L. The frequency and immunodominance of islet-specific CD8+ T-cell responses change after type 1 diabetes diagnosis and treatment. Diabetes (2008) 57(5):1312–20. doi: 10.2337/db07-1594 18305140

[19] Arif S Tree TI Astill TP Tremble JM Bishop AJ Dayan CM. Autoreactive T cell responses show proinflammatory polarization in diabetes but a regulatory phenotype in health. J Clin Invest (2004) 113(3):451–63. doi: 10.1172/JCI19585 PMC324541 14755342

[20] Velthuis JH Unger WW Abreu JR Duinkerken G Franken K Peakman M. Simultaneous detection of circulating autoreactive CD8+ T-cells specific for different islet cell-associated epitopes using combinatorial MHC multimers. Diabetes (2010) 59(7):1721–30. doi: 10.2337/db09-1486 PMC2889772 20357361

[21] Oling V Marttila J Ilonen J Kwok WW Nepom G Knip M. GAD65- and proinsulin-specific CD4+ T-cells detected by MHC class II tetramers in peripheral blood of type 1 diabetes patients and at-risk subjects. J Autoimmun (2005) 25(3):235–43. doi: 10.1016/j.jaut.2005.09.018 16263242

[22] Yang J Danke NA Berger D Reichstetter S Reijonen H Greenbaum C. Islet-specific glucose-6-phosphatase catalytic subunit-related protein-reactive CD4+ T cells in human subjects. J Immunol (2006) 176(5):2781–9. doi: 10.4049/jimmunol.176.5.2781 16493034

[23] Mannering SI Harrison LC Williamson NA Morris JS Thearle DJ Jensen KP. The insulin a-chain epitope recognized by human T cells is posttranslationally modified. J Exp Med (2005) 202(9):1191–7. doi: 10.1084/jem.20051251 PMC2213236 16260488

[24] Skowera A Ladell K McLaren JE Dolton G Matthews KK Gostick E. Beta-cell-specific CD8 T cell phenotype in type 1 diabetes reflects chronic autoantigen exposure. Diabetes (2014) 64(3):916–25. doi: 10.2337/db14-0332 PMC4557541 25249579

[25] Monti P Scirpoli M Rigamonti A Mayr A Jaeger A Bonfanti R. Evidence for *in vivo* primed and expanded autoreactive T cells as a specific feature of patients with type 1 diabetes. J Immunol (2007) 179(9):5785–92. doi: 10.4049/jimmunol.179.9.5785 17947651

[26] Jacobsen LM Bundy BN Greco MN Schatz DA Atkinson MA Brusko TM. Comparing beta cell preservation across clinical trials in recent-onset type 1 diabetes. Diabetes Technol Ther (2020) 22(12):948–53. doi: 10.1089/dia.2020.0305 PMC7757538 32833543

[27] Bluestone JA Buckner JH Herold KC. Immunotherapy: building a bridge to a cure for type 1 diabetes. Science (2021) 373(6554):510–6. doi: 10.1126/science.abh1654 34326232

[28] Jaeckel E Klein L Martin-Orozco N von Boehmer H. Normal incidence of diabetes in NOD mice tolerant to glutamic acid decarboxylase. J Exp Med (2003) 197(12):1635–44. doi: 10.1084/jem.20030215 PMC2193961 12796471

[29] Jaeckel E Lipes MA von Boehmer H. Recessive tolerance to preproinsulin 2 reduces but does not abolish type 1 diabetes. Nat Immunol (2004) 5(10):1028–35. doi: 10.1038/ni1120 15378058

[30] Krishnamurthy B Dudek NL McKenzie MD Purcell AW Brooks AG Gellert S. Responses against islet antigens in NOD mice are prevented by tolerance to proinsulin but not IGRP. J Clin Invest (2006) 116(12):3258–65. doi: 10.1172/JCI29602 PMC1679712 17143333

[31] Selck C Jhala G George DD Kwong C-TJ Christensen MK Pappas E. Extra-islet expression of islet antigen boosts T-cell exhaustion to prevent autoimmune diabetes. bioRxiv (2023). 2023.02.12.528226. doi: 10.1101/2023.02.12.528226 PMC10861925 38285952

[32] Roep BO Solvason N Gottlieb PA Abreu JRF Harrison LC Eisenbarth GS. Plasmid-encoded proinsulin preserves c-peptide while specifically reducing proinsulin-specific CD8(+) T cells in type 1 diabetes. Sci Transl Med (2013) 5(191):191ra82. doi: 10.1126/scitranslmed.3006103 PMC4516024 23803704

[33] Assfalg R Knoop J Hoffman KL Pfirrmann M Zapardiel-Gonzalo JM Hofelich A. Oral insulin immunotherapy in children at risk for type 1 diabetes in a randomised controlled trial. Diabetologia (2021) 64(5):1079–92. doi: 10.1007/s00125-020-05376-1 PMC8012335 33515070

[34] Writing Committee for the Type 1 Diabetes TrialNet Oral Insulin Study G Krischer JP Schatz DA Bundy B Skyler JS Greenbaum CJ. Effect of oral insulin on prevention of diabetes in relatives of patients with type 1 diabetes: a randomized clinical trial. Jama (2017) 318(19):1891–902. doi: 10.1001/jama.2017.17070 PMC5798455 29164254

[35] Alhadj Ali M Liu YF Arif S Tatovic D Shariff H Gibson VB. Metabolic and immune effects of immunotherapy with proinsulin peptide in human new-onset type 1 diabetes. Sci Transl Med (2017) 9(402):eaaf7779. doi: 10.1126/scitranslmed.aaf7779 28794283

[36] Bonifacio E Ziegler AG Klingensmith G Schober E Bingley PJ Rottenkolber M. Effects of high-dose oral insulin on immune responses in children at high risk for type 1 diabetes: the pre-POINT randomized clinical trial. JAMA (2015) 313(15):1541–9. doi: 10.1001/jama.2015.2928 25898052

[37] Vehik K Cuthbertson D Ruhlig H Schatz DA Peakman M Krischer JP. Long-term outcome of individuals treated with oral insulin: diabetes prevention trial-type 1 (DPT-1) oral insulin trial. Diabetes Care (2011) 34(7):1585–90. doi: 10.2337/dc11-0523 PMC3120180 21610124

[38] Fourlanos S Perry C Gellert SA Martinuzzi E Mallone R Butler J. Evidence that nasal insulin induces immune tolerance to insulin in adults with autoimmune diabetes. Diabetes (2011) 60(4):1237–45. doi: 10.2337/db10-1360 PMC3064097 21307076

[39] Ludvigsson J Krisky D Casas R Battelino T Castano L Greening J. GAD65 antigen therapy in recently diagnosed type 1 diabetes mellitus. New Engl J Med (2012) 366(5):433–42. doi: 10.1056/NEJMoa1107096 22296077

[40] Wherrett DK Bundy B Becker DJ DiMeglio LA Gitelman SE Goland R. Antigen-based therapy with glutamic acid decarboxylase (GAD) vaccine in patients with recent-onset type 1 diabetes: a randomised double-blind trial. Lancet (2011) 378(9788):319–27. doi: 10.1016/S0140-6736(11)60895-7 PMC3580128 21714999

[41] Casas R Dietrich F Puente-Marin S Barcenilla H Tavira B Wahlberg J. Intra-lymphatic administration of GAD-alum in type 1 diabetes: long-term follow-up and effect of a late booster dose (the DIAGNODE extension trial). Acta Diabetol (2022) 59(5):687–96. doi: 10.1007/s00592-022-01852-9 PMC8995247 35098372

[42] Tavira B Barcenilla H Wahlberg J Achenbach P Ludvigsson J Casas R. Intralymphatic glutamic acid decarboxylase-alum administration induced Th2-Like-Specific immunomodulation in responder patients: a pilot clinical trial in type 1 diabetes. J Diabetes Res (2018) 2018:9391845. doi: 10.1155/2018/9391845 30009185 PMC5994289

[43] Elding Larsson H Lundgren M Jonsdottir B Cuthbertson D Krischer J Di A-ITSG. Safety and efficacy of autoantigen-specific therapy with 2 doses of alum-formulated glutamate decarboxylase in children with multiple islet autoantibodies and risk for type 1 diabetes: a randomized clinical trial. Pediatr Diabetes (2018) 19(3):410–9. doi: 10.1111/pedi.12611 29171140

[44] Ludvigsson J Faresjo M Hjorth M Axelsson S Cheramy M Pihl M. GAD treatment and insulin secretion in recent-onset type 1 diabetes. New Engl J Med (2008) 359(18):1909–20. doi: 10.1056/NEJMoa0804328 18843118

[45] Harrison LC Honeyman MC Steele CE Stone NL Sarugeri E Bonifacio E. Pancreatic beta-cell function and immune responses to insulin after administration of intranasal insulin to humans at risk for type 1 diabetes. Diabetes Care (2004) 27(10):2348–55. doi: 10.2337/diacare.27.10.2348 15451899

[46] Axelsson S Cheramy M Akerman L Pihl M Ludvigsson J Casas R. Cellular and humoral immune responses in type 1 diabetic patients participating in a phase III GAD-alum intervention trial. Diabetes Care (2013) 36(11):3418–24. doi: 10.2337/dc12-2251 PMC3816912 23863909

[47] Orban T Farkas K Jalahej H Kis J Treszl A Falk B. Autoantigen-specific regulatory T cells induced in patients with type 1 diabetes mellitus by insulin b-chain immunotherapy. J Autoimmun (2010) 34(4):408–15. doi: 10.1016/j.jaut.2009.10.005 PMC2860016 19931408

[48] Greenbaum CJ McCulloch-Olson M Chiu HK Palmer JP Brooks-Worrell B. Parenteral insulin suppresses T cell proliferation to islet antigens. Pediatr Diabetes (2011) 12(3 Pt 1):150–5. doi: 10.1111/j.1399-5448.2010.00674.x PMC2957543 20522167

[49] Thrower SL James L Hall W Green KM Arif S Allen JS. Proinsulin peptide immunotherapy in type 1 diabetes: report of a first-in-man phase I safety study. Clin Exp Immunol (2009) 155(2):156–65. doi: 10.1111/j.1365-2249.2008.03814.x PMC2675245 19040615

[50] Axelsson S Cheramy M Hjorth M Pihl M Akerman L Martinuzzi E. Long-lasting immune responses 4 years after GAD-alum treatment in children with type 1 diabetes. PloS One (2011) 6(12):e29008. doi: 10.1371/journal.pone.0029008 22174945 PMC3236224

[51] Hjorth M Axelsson S Ryden A Faresjo M Ludvigsson J Casas R. GAD-alum treatment induces GAD65-specific CD4+CD25highFOXP3+ cells in type 1 diabetic patients. Clin Immunol (2011) 138(1):117–26. doi: 10.1016/j.clim.2010.10.004 21044870

[52] Ahmed S Cerosaletti K James E Long SA Mannering S Speake C. Standardizing T-cell biomarkers in type 1 diabetes: challenges and recent advances. Diabetes (2019) 68(7):1366–79. doi: 10.2337/db19-0119 PMC6609980 31221801

[53] Nakayama M Abiru N Moriyama H Babaya N Liu E Miao D. Prime role for an insulin epitope in the development of type 1 diabetes in NOD mice. Nature (2005) 435(7039):220–3. doi: 10.1038/nature03523 PMC1364531 15889095

[54] French MB Allison J Cram DS Thomas HE Dempsey-Collier M Silva A. Transgenic expression of mouse proinsulin II prevents diabetes in nonobese diabetic mice. Diabetes (1997) 46(1):34–9. doi: 10.2337/diab.46.1.34 8971078

[55] Kash SF Condie BG Baekkeskov S. Glutamate decarboxylase and GABA in pancreatic islets: lessons from knock-out mice. Horm Metab Res (1999) 31(5):340–4. doi: 10.1055/s-2007-978750 10422732

[56] Kubosaki A Miura J Notkins AL. IA-2 is not required for the development of diabetes in NOD mice. Diabetologia (2004) 47(1):149–50. doi: 10.1007/s00125-003-1252-z 14614561

[57] Oeser JK Parekh VV Wang Y Jegadeesh NK Sarkar SA Wong R. Deletion of the G6pc2 gene encoding the islet-specific glucose-6-phosphatase catalytic subunit-related protein does not affect the progression or incidence of type 1 diabetes in NOD/ShiLtJ mice. Diabetes (2011) 60(11):2922–7. doi: 10.2337/db11-0220 PMC3198073 21896930

[58] Wang J Tsai S Shameli A Yamanouchi J Alkemade G Santamaria P. *In situ* recognition of autoantigen as an essential gatekeeper in autoimmune CD8+ T cell inflammation. Proc Natl Acad Sci U States America (2010) 107(20):9317–22. doi: 10.1073/pnas.0913835107 PMC2889101 20439719

[59] Jhala G Chee J Trivedi PM Selck C Gurzov EN Graham KL. Perinatal tolerance to proinsulin is sufficient to prevent autoimmune diabetes. JCI Insight (2016) 1(10):e86065. doi: 10.1172/jci.insight.86065 27699217 PMC5033903

[60] Tsai S Shameli A Yamanouchi J Clemente-Casares X Wang J Serra P. Reversal of autoimmunity by boosting memory-like autoregulatory T cells. Immunity (2010) 32(4):568–80. doi: 10.1016/j.immuni.2010.03.015 20381385

[61] Buckle I Loaiza Naranjo JD Bergot AS Zhang V Talekar M Steptoe RJ. Tolerance induction by liposomes targeting a single CD8 epitope IGRP(206-214) in a model of type 1 diabetes is impeded by co-targeting a CD4(+) islet epitope. Immunol Cell Biol (2022) 100(1):33–48. doi: 10.1111/imcb.12506 34668580

[62] Le Mercier I Lines JL Noelle RJ. Beyond CTLA-4 and PD-1, the generation z of negative checkpoint regulators. Front Immunol (2015) 6:418. doi: 10.3389/fimmu.2015.00418 26347741 PMC4544156

[63] Okazaki T Chikuma S Iwai Y Fagarasan S Honjo T. A rheostat for immune responses: the unique properties of PD-1 and their advantages for clinical application. Nat Immunol (2013) 14(12):1212–8. doi: 10.1038/ni.2762 24240160

[64] Vignali PDA DePeaux K Watson MJ Ye C Ford BR Lontos K. Hypoxia drives CD39-dependent suppressor function in exhausted T cells to limit antitumor immunity. Nat Immunol (2022) 24(2):267–79. doi: 10.1038/s41590-022-01379-9 PMC10402660 36543958

[65] Cook DP Cunha J Martens PJ Sassi G Mancarella F Ventriglia G. Intestinal delivery of proinsulin and IL-10 via lactococcus lactis combined with low-dose anti-CD3 restores tolerance outside the window of acute type 1 diabetes diagnosis. Front Immunol (2020) 11:1103. doi: 10.3389/fimmu.2020.01103 32582188 PMC7295939

[66] Ejrnaes M Filippi CM Martinic MM Ling EM Togher LM Crotty S. Resolution of a chronic viral infection after interleukin-10 receptor blockade. J Exp Med (2006) 203(11):2461–72. doi: 10.1084/jem.20061462 PMC2118120 17030951

[67] Brooks DG Ha SJ Elsaesser H Sharpe AH Freeman GJ Oldstone MB. IL-10 and PD-L1 operate through distinct pathways to suppress T-cell activity during persistent viral infection. Proc Natl Acad Sci U States America (2008) 105(51):20428–33. doi: 10.1073/pnas.0811139106 PMC2629263 19075244

[68] Ansari MJ Salama AD Chitnis T Smith RN Yagita H Akiba H. The programmed death-1 (PD-1) pathway regulates autoimmune diabetes in nonobese diabetic (NOD) mice. J Exp Med (2003) 198(1):63–9. doi: 10.1084/jem.20022125 PMC2196083 12847137

[69] Galligan A Xu W Fourlanos S Nankervis A Chiang C Mant AM. Diabetes associated with immune checkpoint inhibition: presentation and management challenges. Diabetes Med (2018) 35(9):1283–90. doi: 10.1111/dme.13762 29908076

[70] Stamatouli AM Quandt Z Perdigoto AL Clark PL Kluger H Weiss SA. Collateral damage: insulin-dependent diabetes induced with checkpoint inhibitors. Diabetes (2018) 67(8):1471–80. doi: 10.2337/dbi18-0002 PMC6054443 29937434

[71] Grebinoski S Zhang Q Cillo AR Manne S Xiao H Brunazzi EA. Autoreactive CD8(+) T cells are restrained by an exhaustion-like program that is maintained by LAG3. Nat Immunol (2022) 23(6):868–77. doi: 10.1038/s41590-022-01210-5 PMC9179227 35618829

[72] Ciecko AE Schauder DM Foda B Petrova G Kasmani MY Burns R. Self-renewing islet TCF1(+) CD8 T cells undergo IL-27-Controlled differentiation to become TCF1(-) terminal effectors during the progression of type 1 diabetes. J Immunol (2021) 207(8):1990–2004. doi: 10.4049/jimmunol.2100362 34507949 PMC8492517

[73] Zakharov PN Hu H Wan X Unanue ER. Single-cell RNA sequencing of murine islets shows high cellular complexity at all stages of autoimmune diabetes. J Exp Med (2020) 217(6):e20192362. doi: 10.1084/jem.20192362 32251514 PMC7971127

[74] McKinney EF Lee JC Jayne DR Lyons PA Smith KG. T-Cell exhaustion, co-stimulation and clinical outcome in autoimmunity and infection. Nature (2015) 523(7562):612–6. doi: 10.1038/nature14468 PMC4623162 26123020

[75] Diggins KE Serti E Muir V Rosasco M Lu T Balmas E. Exhausted-like CD8+ T cell phenotypes linked to c-peptide preservation in alefacept-treated T1D subjects. JCI Insight (2021) 6(3):e142680. doi: 10.1172/jci.insight.142680 33351781 PMC7934874

[76] Wiedeman AE Muir VS Rosasco MG DeBerg HA Presnell S Haas B. Autoreactive CD8+ T cell exhaustion distinguishes subjects with slow type 1 diabetes progression. J Clin Invest (2020) 130(1):480–90. doi: 10.1172/JCI126595 PMC6934185 31815738

[77] Pescovitz MD Greenbaum CJ Bundy B Becker DJ Gitelman SE Goland R. B-lymphocyte depletion with rituximab and beta-cell function: two-year results. Diabetes Care (2014) 37(2):453–9. doi: 10.2337/dc13-0626 PMC3898764 24026563

[78] Pescovitz MD Greenbaum CJ Krause-Steinrauf H Becker DJ Gitelman SE Goland R. Rituximab, b-lymphocyte depletion, and preservation of beta-cell function. New Engl J Med (2009) 361(22):2143–52. doi: 10.1056/NEJMoa0904452 PMC6410357 19940299

[79] Orban T Bundy B Becker DJ Dimeglio LA Gitelman SE Goland R. Costimulation modulation with abatacept in patients with recent-onset type 1 diabetes: follow-up 1 year after cessation of treatment. Diabetes Care (2014) 37(4):1069–75. doi: 10.2337/dc13-0604 PMC3964491 24296850

[80] Orban T Bundy B Becker DJ DiMeglio LA Gitelman SE Goland R. Co-Stimulation modulation with abatacept in patients with recent-onset type 1 diabetes: a randomised, double-blind, placebo-controlled trial. Lancet (2011) 378(9789):412–9. doi: 10.1016/S0140-6736(11)60886-6 PMC3462593 21719096

[81] Haller MJ Gitelman SE Gottlieb PA Michels AW Rosenthal SM Shuster JJ. Anti-thymocyte globulin/G-CSF treatment preserves beta cell function in patients with established type 1 diabetes. J Clin Invest (2015) 125(1):448–55. doi: 10.1172/JCI78492 PMC4382237 25500887

[82] Herold KC Hagopian W Auger JA Poumian-Ruiz E Taylor L Donaldson D. Anti-CD3 monoclonal antibody in new-onset type 1 diabetes mellitus. New Engl J Med (2002) 346(22):1692–8. doi: 10.1056/NEJMoa012864 12037148

[83] Keymeulen B van Maurik A Inman D Oliveira J McLaughlin R Gittelman RM. A randomised, single-blind, placebo-controlled, dose-finding safety and tolerability study of the anti-CD3 monoclonal antibody otelixizumab in new-onset type 1 diabetes. Diabetologia (2021) 64(2):313–24. doi: 10.1007/s00125-020-05317-y PMC7801303 33145642

[84] Keymeulen B Vandemeulebroucke E Ziegler AG Mathieu C Kaufman L Hale G. Insulin needs after CD3-antibody therapy in new-onset type 1 diabetes. New Engl J Med (2005) 352(25):2598–608. doi: 10.1056/NEJMoa043980 15972866

[85] Herold KC Bundy BN Long SA Bluestone JA DiMeglio LA Dufort MJ. An anti-CD3 antibody, teplizumab, in relatives at risk for type 1 diabetes. New Engl J Med (2019) 381(7):603–13. doi: 10.1056/NEJMoa1902226 PMC6776880 31180194

[86] Rigby MR DiMeglio LA Rendell MS Felner EI Dostou JM Gitelman SE. Targeting of memory T cells with alefacept in new-onset type 1 diabetes (T1DAL study): 12 month results of a randomised, double-blind, placebo-controlled phase 2 trial. Lancet Diabetes Endocrinol (2013) 1(4):284–94. doi: 10.1016/S2213-8587(13)70111-6 PMC3957186 24622414

[87] Rigby MR Harris KM Pinckney A DiMeglio LA Rendell MS Felner EI. Alefacept provides sustained clinical and immunological effects in new-onset type 1 diabetes patients. J Clin Invest (2015) 125(8):3285–96. doi: 10.1172/JCI81722 PMC4623571 26193635

[88] Quattrin T Haller MJ Steck AK Felner EI Li Y Xia Y. Golimumab and beta-cell function in youth with new-onset type 1 diabetes. New Engl J Med (2020) 383(21):2007–17. doi: 10.1056/NEJMoa2006136 33207093

[89] Mastrandrea L Yu J Behrens T Buchlis J Albini C Fourtner S. Etanercept treatment in children with new-onset type 1 diabetes: pilot randomized, placebo-controlled, double-blind study. Diabetes Care (2009) 32(7):1244–9. doi: 10.2337/dc09-0054 PMC2699714 19366957

[90] von Herrath M Bain SC Bode B Clausen JO Coppieters K Gaysina L. Anti-interleukin-21 antibody and liraglutide for the preservation of beta-cell function in adults with recent-onset type 1 diabetes: a randomised, double-blind, placebo-controlled, phase 2 trial. Lancet Diabetes Endocrinol (2021) 9(4):212–24. doi: 10.1016/S2213-8587(21)00019-X 33662334

[91] Greenbaum CJ Serti E Lambert K Weiner LJ Kanaparthi S Lord S. IL-6 receptor blockade does not slow beta cell loss in new-onset type 1 diabetes. JCI Insight (2021) 6(21):e150074. doi: 10.1172/jci.insight.150074 34747368 PMC8663550

[92] Moran A Bundy B Becker DJ DiMeglio LA Gitelman SE Goland R. Nterleukin-1 antagonism in type 1 diabetes of recent onset: two multicentre, randomised, double-blind, placebo-controlled trials. Lancet (2013) 381(9881):1905–15. doi: 10.1016/S0140-6736(13)60023-9 PMC3827771 23562090

[93] Gitelman SE Gottlieb PA Felner EI Willi SM Fisher LK Moran A. Antithymocyte globulin therapy for patients with recent-onset type 1 diabetes: 2 year results of a randomised trial. Diabetologia (2016) 59(6):1153–61. doi: 10.1007/s00125-016-3917-4 PMC4869699 27053235

[94] Hagopian W Ferry RJ Jr. Sherry N Carlin D Bonvini E Johnson S. Teplizumab preserves c-peptide in recent-onset type 1 diabetes: two-year results from the randomized, placebo-controlled protege trial. Diabetes (2013) 62(11):3901–8. doi: 10.2337/db13-0236 PMC3806608 23801579

[95] Herold KC Gitelman SE Ehlers MR Gottlieb PA Greenbaum CJ Hagopian W. Teplizumab (anti-CD3 mAb) treatment preserves c-peptide responses in patients with new-onset type 1 diabetes in a randomized controlled trial: metabolic and immunologic features at baseline identify a subgroup of responders. Diabetes (2013) 62(11):3766–74. doi: 10.2337/db13-0345 PMC3806618 23835333

[96] Perdigoto AL Preston-Hurlburt P Clark P Long SA Linsley PS Harris KM. Treatment of type 1 diabetes with teplizumab: clinical and immunological follow-up after 7 years from diagnosis. Diabetologia (2019) 62(4):655–64. doi: 10.1007/s00125-018-4786-9 PMC6402971 30569273

[97] Long SA Rieck M Sanda S Bollyky JB Samuels PL Goland R. Rapamycin/IL-2 combination therapy in patients with type 1 diabetes augments tregs yet transiently impairs beta-cell function. Diabetes (2012) 61(9):2340–8. doi: 10.2337/db12-0049 PMC3425404 22721971

[98] Odegard JM Nepom GT Wambre E. Biomarkers for antigen immunotherapy in allergy and type 1 diabetes. Clin Immunol (2015) 161(1):44–50. doi: 10.1016/j.clim.2015.05.023 26122171 PMC4628579

[99] Yang JHM Ward-Hartstonge KA Perry DJ Blanchfield JL Posgai AL Wiedeman AE. Guidelines for standardizing T-cell cytometry assays to link biomarkers, mechanisms, and disease outcomes in type 1 diabetes. Eur J Immunol (2022) 52(3):372–88. doi: 10.1002/eji.202049067 PMC9006584 35025103

[100] Mannering SI Morris JS Jensen KP Purcell AW Honeyman MC van Endert PM. A sensitive method for detecting proliferation of rare autoantigen-specific human T cells. J Immunol Methods (2003) 283(1-2):173–83. doi: 10.1016/j.jim.2003.09.004 14659909

[101] Yeo L Woodwyk A Sood S Lorenc A Eichmann M Pujol-Autonell I. Autoreactive T effector memory differentiation mirrors beta cell function in type 1 diabetes. J Clin Invest (2018) 128(8):3460–74. doi: 10.1172/JCI120555 PMC6063477 29851415

[102] Roep BO Kleijwegt FS van Halteren AG Bonato V Boggi U Vendrame F. Islet inflammation and CXCL10 in recent-onset type 1 diabetes. Clin Exp Immunol (2010) 159(3):338–43. doi: 10.1111/j.1365-2249.2009.04087.x PMC2819499 20059481

[103] Uno S Imagawa A Saisho K Okita K Iwahashi H Hanafusa T. Expression of chemokines, CXC chemokine ligand 10 (CXCL10) and CXCR3 in the inflamed islets of patients with recent-onset autoimmune type 1 diabetes. Endocr J (2010) 57(11):991–6. doi: 10.1507/endocrj.K10E-076 20966598

[104] Tanaka S Nishida Y Aida K Maruyama T Shimada A Suzuki M. Enterovirus infection, CXC chemokine ligand 10 (CXCL10), and CXCR3 circuit: a mechanism of accelerated beta-cell failure in fulminant type 1 diabetes. Diabetes (2009) 58(10):2285–91. doi: 10.2337/db09-0091 PMC2750208 19641142

[105] Damond N Engler S Zanotelli VRT Schapiro D Wasserfall CH Kusmartseva I. A map of human type 1 diabetes progression by imaging mass cytometry. Cell Metab (2019) 29(3):755–68 e5. doi: 10.1016/j.cmet.2018.11.014 30713109 PMC6821395

[106] Wang YJ Traum D Schug J Gao L Liu C Consortium H. Multiplexed *In situ* imaging mass cytometry analysis of the human endocrine pancreas and immune system in type 1 diabetes. Cell Metab (2019) 29(3):769–83 e4. doi: 10.1016/j.cmet.2019.01.003 30713110 PMC6436557

[107] Ma KY Schonnesen AA He C Xia AY Sun E Chen E. High-throughput and high-dimensional single-cell analysis of antigen-specific CD8(+) T cells. Nat Immunol (2021) 22(12):1590–8. doi: 10.1038/s41590-021-01073-2 PMC9184244 34811538

[108] Stoeckius M Hafemeister C Stephenson W Houck-Loomis B Chattopadhyay PK Swerdlow H. Simultaneous epitope and transcriptome measurement in single cells. Nat Methods (2017) 14(9):865–8. doi: 10.1038/nmeth.4380 PMC5669064 28759029

[109] Mallajosyula V Ganjavi C Chakraborty S McSween AM Pavlovitch-Bedzyk AJ Wilhelmy J. CD8(+) T cells specific for conserved coronavirus epitopes correlate with milder disease in COVID-19 patients. Sci Immunol (2021) 6(61):eabg5669. doi: 10.1126/sciimmunol.abg5669 34210785 PMC8975171

